# Supplementary material for: Evaluation of the immunogenicity of prime-boost vaccination with the replication-deficient viral vectored COVID-19 vaccine candidate ChAdOx1 nCoV-19
Source: NPJ Vaccines. 2020 Jul 27;5:69. doi: 10.1038/s41541-020-00221-3 (PMC7385486; doi:10.1038/s41541-020-00221-3)
Supplement: Supplementary file 1 — Supplementary Information [file 41541_2020_221_MOESM1_ESM.pdf]

A. Spike peptide stimulated splenocytes

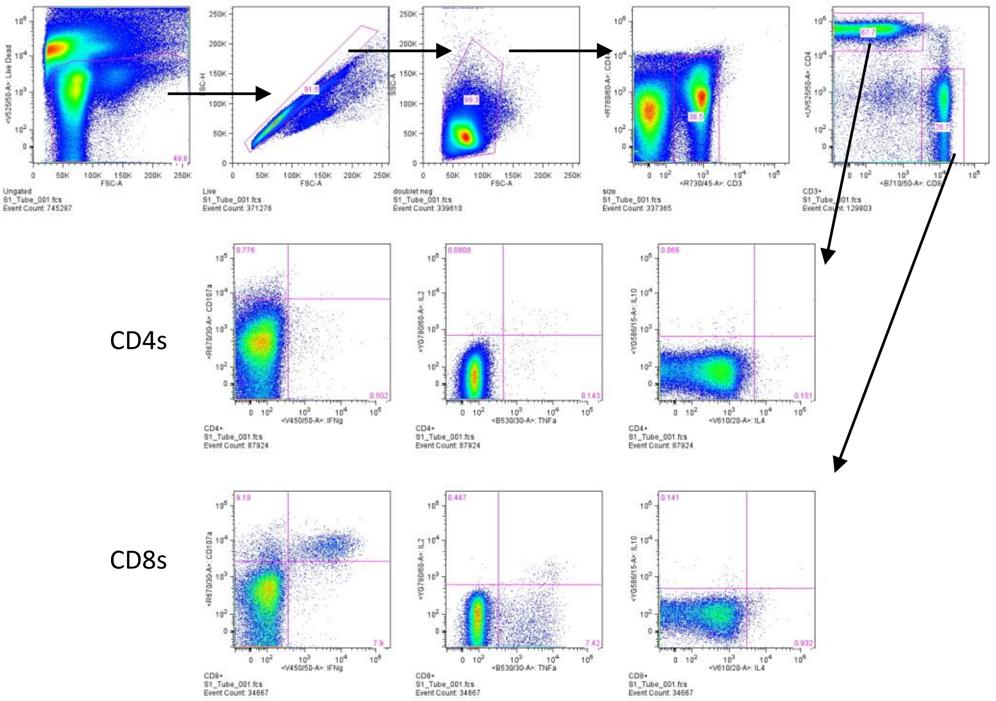

B. Media stimulated splenocytes

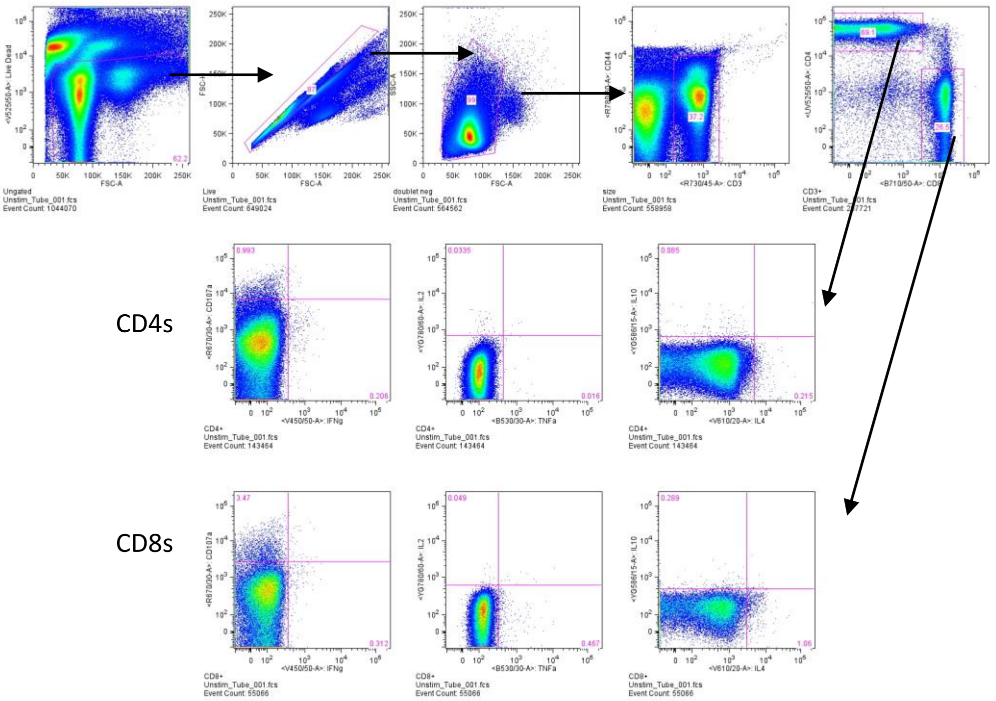

Supplementary Figure 1. Illustration of gating strategy to assess cytokine responses of SARS-CoV-2 S peptide (A) and media (B) stimulated murine splenocytes.

A. Spike peptide stimulated PBMC

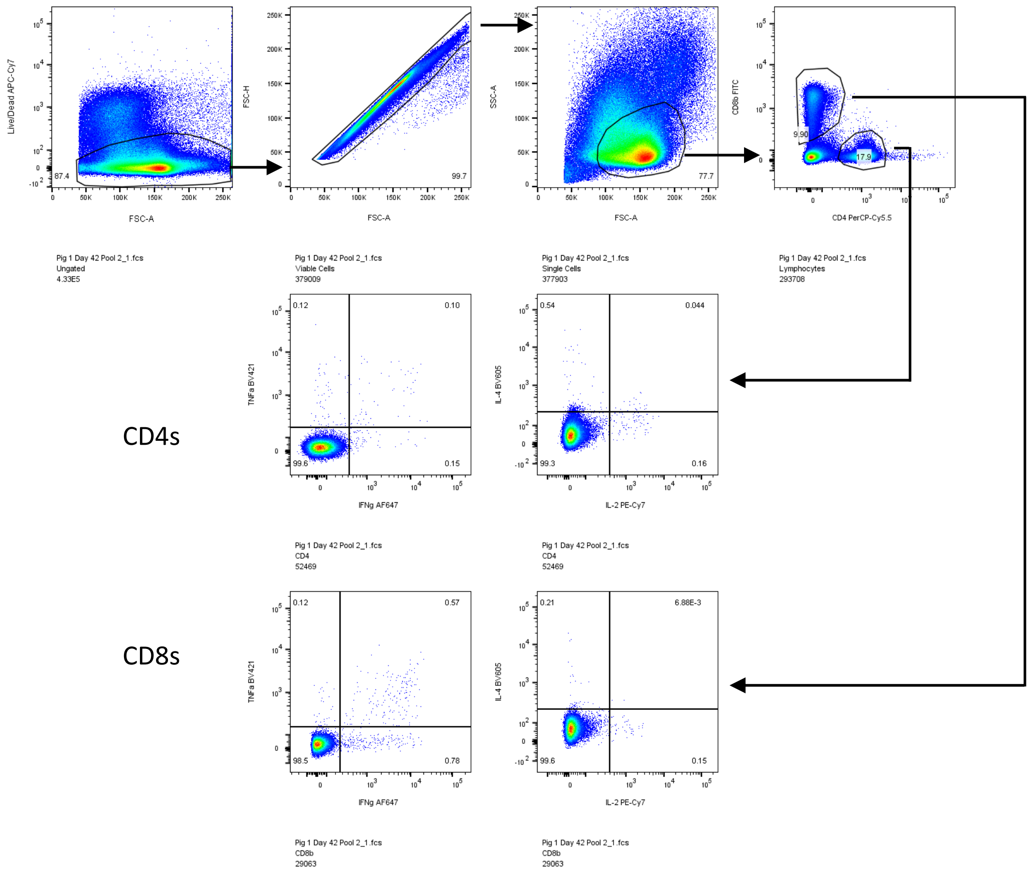

B. Media stimulated PBMC

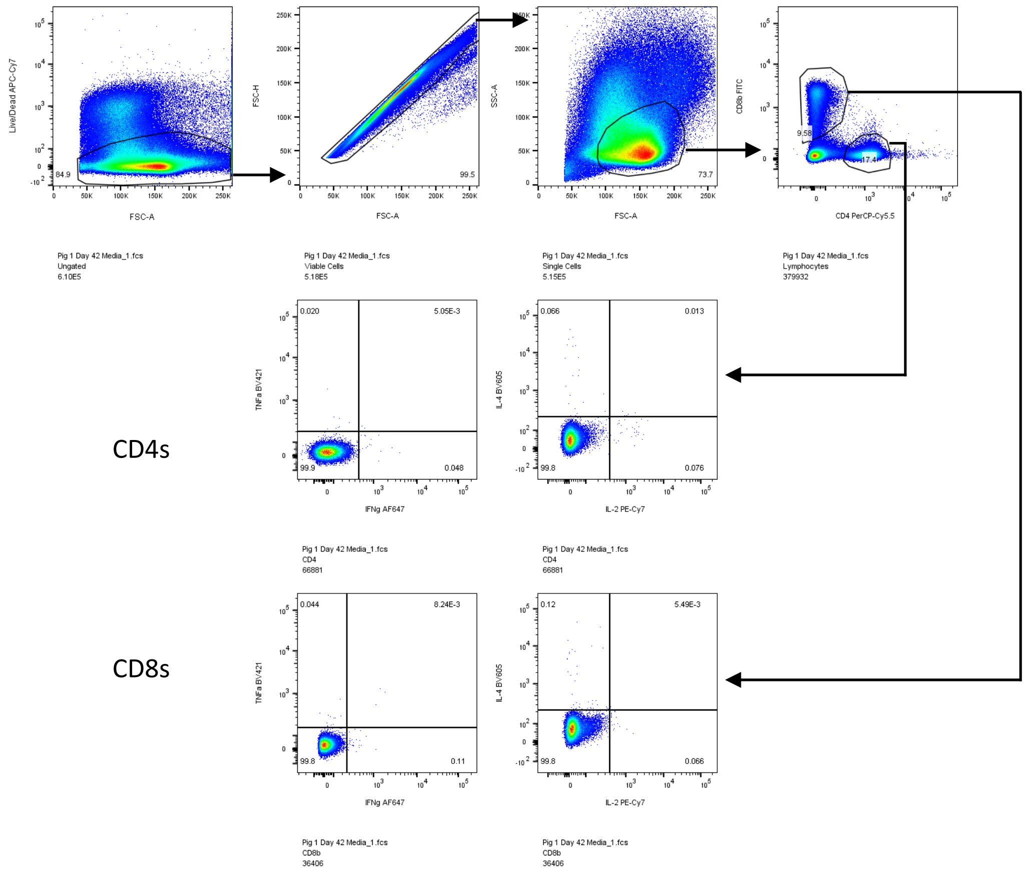

Supplementary Figure 2. Illustration of gating strategy to assess cytokine responses of SARS-CoV-2 S peptide (A) and media (B) stimulated porcine PBMC.
