# Supplementary material for: Evaluation of the immunogenicity of prime-boost vaccination with the replication-deficient viral vectored COVID-19 vaccine candidate ChAdOx1 nCoV-19
Source: NPJ Vaccines. 2020 Jul 27;5:69. doi: 10.1038/s41541-020-00221-3 (PMC7385486; doi:10.1038/s41541-020-00221-3)
Supplement: Supplementary file 2 — Reporting Summary [file 41541_2020_221_MOESM2_ESM.pdf]

nature research

Corresponding author(s): Simon Paul Graham
Last updated by author(s): Jul 8, 2020

Reporting Summary

Nature Research wishes to improve the reproducibility of the work that we publish. This form provides structure for consistency and transparency in reporting. For further information on Nature Research policies, see our Editorial Policies and the Editorial Policy Checklist.

Statistics

For all statistical analyses, confirm that the following items are present in the figure legend, table legend, main text, or Methods section.

n/a	Confirmed	
☐	☒	The exact sample size (*n*) for each experimental group/condition, given as a discrete number and unit of measurement
☐	☒	A statement on whether measurements were taken from distinct samples or whether the same sample was measured repeatedly
☐	☒	The statistical test(s) used AND whether they are one- or two-sided<br>*Only common tests should be described solely by name; describe more complex techniques in the Methods section.*
☒	☐	A description of all covariates tested
☐	☒	A description of any assumptions or corrections, such as tests of normality and adjustment for multiple comparisons
☐	☒	A full description of the statistical parameters including central tendency (e.g. means) or other basic estimates (e.g. regression coefficient) AND variation (e.g. standard deviation) or associated estimates of uncertainty (e.g. confidence intervals)
☐	☒	For null hypothesis testing, the test statistic (e.g. *F*, *t*, *r*) with confidence intervals, effect sizes, degrees of freedom and *P* value noted<br>*Give P values as exact values whenever suitable.*
☒	☐	For Bayesian analysis, information on the choice of priors and Markov chain Monte Carlo settings
☒	☐	For hierarchical and complex designs, identification of the appropriate level for tests and full reporting of outcomes
☒	☐	Estimates of effect sizes (e.g. Cohen's *d*, Pearson's *r*), indicating how they were calculated

Our web collection on statistics for biologists contains articles on many of the points above.

Software and code

Policy information about availability of computer code

Data collection	No software was used
Data analysis	GraphPad Prism v8.1.2; FlowJo v9; FlowJo X

For manuscripts utilizing custom algorithms or software that are central to the research but not yet described in published literature, software must be made available to editors and reviewers. We strongly encourage code deposition in a community repository (e.g. GitHub). See the Nature Research guidelines for submitting code & software for further information.

Data

Policy information about availability of data

All manuscripts must include a data availability statement. This statement should provide the following information, where applicable:
- Accession codes, unique identifiers, or web links for publicly available datasets
- A list of figures that have associated raw data
- A description of any restrictions on data availability

The data that support the findings of this study are available from the corresponding authors upon reasonable request.

Field-specific reporting

Life sciences study design

All studies must disclose on these points even when the disclosure is negative.

Sample size	Sample size used for the mouse experiment was based on a power calculation utilizing prior immunogenicity data from these strains of mice. The sample size for the pig study was not based on a power calculation and since this was the first study to evaluate COVID-19 vaccine candidates in this species, a small group size was appropriate from an ethical perspective.
Data exclusions	No data were excluded.
Replication	Data were not replicated.
Randomization	Mice and pigs were randomly allocated to treatment groups.
Blinding	Blinding was not applied.

Reporting for specific materials, systems and methods

We require information from authors about some types of materials, experimental systems and methods used in many studies. Here, indicate whether each material, system or method listed is relevant to your study. If you are not sure if a list item applies to your research, read the appropriate section before selecting a response.

Materials & experimental systems

n/a	Involved in the study	
☐	☒	Antibodies
☐	☒	Eukaryotic cell lines
☒	☐	Palaeontology and archaeology
☐	☒	Animals and other organisms
☒	☐	Human research participants
☒	☐	Clinical data
☒	☐	Dual use research of concern

Methods

n/a	Involved in the study	
☒	☐	ChIP-seq
☐	☒	Flow cytometry
☒	☐	MRI-based neuroimaging

Antibodies

Antibodies used	For mouse flow cytometry experiments the following antibodies were used: anti-mouse TNFa-A488 (Clone MP6-XT22, Biolegend, Catalogue Number 506313), anti-mouse CD8 PerCPCy5.5 (Clone 53-6.7, Thermofisher Cat Number 45-0081-82), anti-mouse IFNg eFluro450 (Clone XMG1.2, Thermofisher Cat Number 48-7311-82), anti-mouse IL-4 BV605 (Clone 11B11, Catalogue Number 504126), anti-mouse CD127 BV650 (Clone A7R34, Biolegend Catalogue Number 135043), anti-mouse CD62L BV711 (Clone MEL-14, Biolegend Catalogue Number 104445), anti-mouse CD107a Alexa647 (Clone 1DB4, Biolegend Catalogue Number 121610), anti-mouse CD3 (Clone 500A2, BD Catalogue Number 557984), anti-mouse CD44 APCCy7 (Clone IM7, Biolegend Catalogue Number 103028), anti-mouse IL-10 (Clone JES5-16E3, BD Catalogue Number 505008), anti-mouse IL-2 PECy7 (Clone JES6-5H4, Thermofisher Catalogue Number 25-7021-82), anti-mouse CD4 BUV496 (Clone GK1.5, BD Catalogue Number 564667).<br>For porcine flow cytometry experiments, the following antibodies were used: Anti-pig CD4α PerCP-Cy5.5 (Clone 74-12-4, BD, Catalogue Number 561474), Anti-pig CD8β FITC (ClonePPT23, Bio-Rad, Catalogue Number MCA5954F), Anti-human TNF-α BV421 (clone Mab11, BioLegend, Catalogue Number 502932), Anti-bovine IFN-γ AF647 (Clone CC302, Bio-Rad, Catalogue Number MCA1783A647), Anti-human IL-4 BV605 (Clone MP4-25D2, BioLegend, Catalogue Number 500827), Anti-pig IL-2 Purified (Clone A150D 3F1 2H2, ThermoFisher Scientific, Catalogue Number ASC0924), Anti-mouse IgG2a PE Cy7 (Clone RMG2a-62, BioLegend, Catalogue Number 407114).
Validation	All mouse antibodies were validated for use with mouse samples by the supplier. All antibodies used for porcine flow cytometry were validated for use with pig samples by the supplier.

Eukaryotic cell lines

Policy information about cell lines

Cell line source(s)	Human Embryonic Kidney 293T (HEK293T) were purchased from ATCC. Vero E6 cells were obtained from the Central Services Unit at The Pirbright Institute, UK.
Authentication	HEK293T cells and Vero E6 cells were not further authenticated.
Mycoplasma contamination	All cell lines tested negative for Mycoplasma spp. Vero E6 cells - MycoAlert Mycoplasma Detection Kit, Lonza Cat. # LT07-318; HEK293T cells - MycoAlert Plus Kit, Lonza Cat. # LT07-703.

Commonly misidentified lines (See ICLAC register)	No commonly misidentified cell lines were used in this study.

Animals and other organisms

Policy information about studies involving animals; ARRIVE guidelines recommended for reporting animal research

Laboratory animals	Inbred female BALB/cOlaHsd (BALB/c) and outbred Crl:CD1 (CD1) mice were purchase from commercial suppliers (Envigo and Charles River Laboratories, respectively) and randomly allocated into 'prime-only' or 'prime-boost' vaccination groups (BALB/c n=5 and CD1 n=8) upon arrival. Mice were 9-10 weeks of age at the start of the experiment. 8–10-week-old, weaned, female, Large White-Landrace-Hampshire cross-bred pigs from purchased from a commercial rearing unit.
Wild animals	The study did not involve wild animals.
Field-collected samples	The study did not involve samples collected from the field.
Ethics oversight	Studies were performed in accordance with the UK Animals (Scientific Procedures) Act 1986 and with approval from the relevant local Animal Welfare and Ethical Review Body (AWERB) (Mice - Project License P9808B4F1 and University of Oxford AWERB, and pigs - Project License PP1804248 and The Pirbright Institute AWERB).

Note that full information on the approval of the study protocol must also be provided in the manuscript.

Flow Cytometry

Plots

Confirm that:

- [x] The axis labels state the marker and fluorochrome used (e.g. CD4-FITC).
- [x] The axis scales are clearly visible. Include numbers along axes only for bottom left plot of group (a 'group' is an analysis of identical markers).
- [x] All plots are contour plots with outliers or pseudocolor plots.
- [x] A numerical value for number of cells or percentage (with statistics) is provided.

Methodology

Sample preparation	Single cell suspension of murine splenocytes were prepared by passing cells through 70µM cell strainers and ACK lysis prior to resuspension in complete medium. Cells were stimulated at 37oC for 6 hours with 2µg/ml S1 or S2 pools of peptide, media or cell stimulation cocktail (containing PMA-Ionomycin, Biolegend), together with 1µg/ml Golgi-plug (BD) with the addition of 2µl/ml CD107a-Alexa647).<br>Porcine PBMC were isolated from heparinised blood by density gradient centrifugation and cryopreserved in cold 10% DMSO (Sigma-Aldrich) in HI FBS. Resuscitated PBMC were suspended in RPMI 1640 medium, GlutaMAX supplement, HEPES (Gibco) supplemented with 10 % HI FBS (New Zealand origin, Life Science Production, Bedford, UK), 1% Penicillin-Streptomycin and 0.1% 2-mercaptoethanol (50 mM; Gibco) (cRPMI). suspended in cRPMI at a density of 2 × 107 cells/mL and added to 50 µL/ well to 96-well round bottom plates. PBMCs were stimulated in triplicate wells with the SARS-CoV-2 S peptide pools (1 µg/ mL/peptide). Unstimulated cells in triplicate wells were used as a negative control. After 14 hours incubation at 37 °C, 5% CO2, cytokine secretion was blocked by addition 1:1,000 BD GolgiPlug (BD Biosciences) and cells were further incubated for 6 hours. PBMC were washed in PBS prior to staining.
Instrument	BD FortessaX2 (mouse) and BD LSRFortessa (Pig)
Software	FACS DIVA software
Cell population abundance	For mouse samples, an an acquisition threshold was set at a minimum of 5000 events in the live CD3+ gate.<br>For pig samples, an acquisition threshold was set at a minimum of 100,000 events in the live gate.
Gating strategy	For mouse samples, antigen specific T cells were identified by gating on LIVE/DEAD negative, doublet negative (FSC-H vs FSC-A), size (FSC-H vs SSC), CD3+, CD4+ or CD8+ cells and cytokine positive. Cytokine positive responses are presented after subtraction of the background response detected in the corresponding unstimulated sample (media containing CD107a and Golgi-plug) of each individual spleen sample.<br>For pig samples, antigen specific T cells were identified by gating on LIVE/DEAD negative, doublet negative (FSC-H vs FSC-A), size (FSC-H vs SSC), CD4+ or CD8b+ cells and cytokine positive. Cytokine positive responses are presented after subtraction of the background response detected in the corresponding unstimulated sample (media and Golgi-plug) of each individual PBMC sample.

- [x] Tick this box to confirm that a figure exemplifying the gating strategy is provided in the Supplementary Information.
